# Myopathy after rapid correction of hyperthyroidism: A case report and review of literature

**DOI:** 10.1097/MD.0000000000018878

**Published:** 2020-01-17

**Authors:** Ran Lu, Haining Wang, Tianpei Hong, Hongwei Gao

**Affiliations:** Department of Endocrinology and Metabolism, Peking University Third Hospital, Beijing, China.

**Keywords:** creatine kinase, hyperthyroidism, myalgia, myopathy, relative hypothyroidism

## Abstract

**Rationale:**

Myalgia and elevated creatine kinase (CK) have been reported during the treatment of hyperthyroid patients. The causes of these symptoms are usually considered to be treatments of antithyroid drugs (ATDs), thyroidectomy or radio-iodine (131-I). However, the underlying cause may be the rapid correction of thyrotoxicosis (or relative hypothyroidism), which was usually neglected in clinical practice.

**Patient concerns:**

This report describes a case of a 25-year-old female with typical symptoms and laboratory test results of Grave hyperthyroidism. The patient complained about fatigue and myalgia 7 weeks after receiving methimazole (MMI) treatment. Blood tests showed dramatically elevated serum CK level, although free triiodothyronine (FT3) and free thyroxine (FT4) level had returned to the normal reference range. MMI was; therefore, discontinued and the patient's muscular symptoms disappeared quickly with the normalization of CK level and the relapse of hyperthyroidism. Later she received 131-I treatment and suffered similar muscular symptoms when FT3 and FT4 decreased to the normal range. This time, her symptoms were quickly relieved by levothyroxine (L-T4) replacement treatment.

**Diagnoses:**

Myopathy induced by rapid correction of hyperthyroidism (or relative hypothyroidism).

**Interventions:**

MMI was discontinued after the patient's first episode of muscular symptoms. And for her second episode of muscular injury after 131-I treatment, we initiated L-T4 supplementation.

**Outcomes:**

For the 2 episodes of muscular injury after ATDs or 131-I treatment, both of the interventions mentioned above brought a rapid relief of symptoms accompanied with normalization of CK level and restoration of thyroid hormone level.

**Lessons:**

Myopathy can be caused by a rapid reduction of thyroid hormone during the treatment of hyperthyroidism. This relative hypothyroidism syndrome should be considered if patients make complaints about fatigue and myalgia, even when thyroid hormone level is within the normal range during the antithyroid treatments. Serum CK level and thyroid function should be closely monitored post antithyroid treatments. Reduction of ATD dosage or replacement of thyroid hormone is suggested to relieve muscular symptoms.

## 1 Introduction

Newly-onset musculoskeletal symptoms (such as fatigue and myalgia) with elevated creatine kinase (CK) have been reported during the treatment of hyperthyroidism in patients without obvious evidence of hypothyroidism from treatment. These symptoms are reported to result from treatments including antithyroid drugs (ATDs),^[1]^ thyroidectomy^[2]^ or radio-iodine (131-I).^[3]^ However, the underlying reason for the muscular symptoms may be the rapid reduction of thyrotoxicosis (or relative hypothyroidism) by antithyroid treatments. Although several researchers hypothesized that relative hypothyroidism during antithyroid treatment could induce myopathy that is similar to the symptoms observed in hypothyroidism, there was no definitive data to support this theory.

Here we report a case in which the patient received ATDs and 131-I treatments respectively, and developed muscular symptoms after each of the 2 treatments. In both cases, we observed elevated muscle enzymes with the rapid normalization of thyroid hormone levels. This particular case can strongly support the idea that “relative hypothyroidism” can induce myopathy during the treatment of hyperthyroidism.

## 2 Case report

The informed consent for a case report was obtained from the patient. A 25-year old female visited our outpatient clinic on October 28, 2015, and complained intermittent palpitations, hand tremor, heat intolerance, mild fatigue, and rapid weight loss. She did not have muscular pain, nausea, ophthalmalgia, or lower limb edema. On physical examination, she was found with accelerated heart rate, grade II goiter, and exophthalmos. Blood tests showed decreased thyrotropin (TSH <0.008 μU/mL, normal 0.55–4.78), increased free thyroxine (FT_4_, 4.5 ng/dL, normal 0.89–1.80) and free triiodothyronine (FT_3_, 15.0 pg/mL, normal 2.3–4.2), positive TSH receptor antibody (3.55U/L, normal <1.75), negative thyroid peroxidase antibody and thyroglobulin antibody. Based on her clinical symptoms and laboratory test results, the patient was diagnosed with Grave disease. She was initially treated with methimazole (MMI) 10 mg twice a day, and the dose of MMI was increased to 15 mg twice a day 2 weeks later. Seven weeks after treatment, the patient felt improvements in most of the aforementioned symptoms. However, she complained muscular fatigue, and pain in the neck, shoulder and upper limbs areas and those symptoms were aggravated after exertion. Physical examination suggested mild weakness of limbs. Blood tests showed improvement in thyroid function with FT_4_ 1.28 ng/dL, FT_3_ 3.9 pg/mL, and TSH 0.02 μIU/mL; however, CK was dramatically elevated to 1094 U/L (reference level: 26–140 U/L). Other tests results including complete blood count (CBC), liver enzyme, renal function, coagulation function, serum myoglobin and electrolytes (including serum potassium) were within the normal range. The antinuclear antibodies (ANA) were negative with erythrocyte sedimentation rate (ESR) and C-reactive protein (CRP) within the normal range. Since the potential induction of muscular damage by ATDs could not be excluded, the MMI was discontinued. Only 1 week later, the patient reported significant improvement in myalgia and muscle weakness. These were in accordance with a normal level of serum CK level (64 U/L), while the thyroid function tests also suggested the relapse of hyperthyroidism (FT_3_ 13.37 pg/mL, FT_4_ 3.70 ng/dL, TSH <0.01 uIU/mL) following termination of MMI.

**Figure 1:**
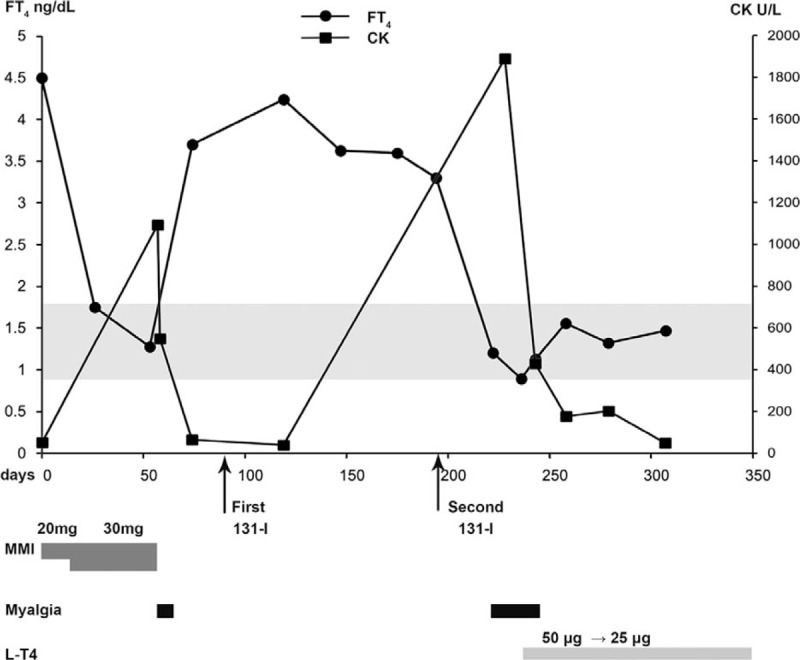
Clinical course of the reported case. Prescription, muscular symptoms, and serum levels of thyroxine and creatine kinase are indicated. 131-I = radio-iodine therapy, CK = creatine kinase, FT4 = free thyroxine, MMI = methimazole.

As a result, the patient was recommended to receive radioiodine therapy. She received twice 131-I treatment at the doses of 5 mCi and 3.5 mCi on January 26, 2016 and May 10, 2016, respectively. Nearly 1 month after the second 131-I treatment, the patient again experienced muscular weakness and pain, with the nature and location as observed before. Blood tests showed that thyroid function was close to normal range (FT_3_ 3.21 pg/mL, FT_4_ 1.2 ng/dL, TSH 0.01 uIU/mL) and significantly elevated serum CK level (1890 U/L). Other tests results including CBC, liver enzyme, renal function, coagulation function, serum myoglobin, and electrolytes were within the normal range. At this time, we are convinced that the patient's muscular damage was caused by a rapid decline in thyroid hormone level and initiated the treatment of levothyroxine (L-T4) 50 μg daily. The patient's muscular symptoms disappeared within 1 week with normal CK level. Later the dosage of L-T4 was tittered to 25 μg daily according to her thyroid function. The clinical course of this case was shown in Figure 1.

## 3 Discussion

Neuromuscular manifestations are common in both hyper- and hypothyroid disease. Muscle weakness and wasting are common clinical manifestations of thyrotoxicosis.^[4]^ This is termed thyrotoxic myopathy, and is usually presented as gradual onset of weakness and fatigue without myalgias, along with a normal or even decreased CK level.^[5]^ Thyrotoxic periodic paralysis is another common complication that is usually seen in Asian men after exercise. It is not associated with myalgias, and manifests with normal CK.^[6]^ There are only 4 cases reporting hyperthyroidism-induced rhabdomyolysis published in the medical literature.^[7]^ Hypothyroid patients, on the other hand, often complain of articular and muscular pains, and may even present with joint effusions involving knees or small joints. Hypothyroidism has also been clearly associated with pathologic alterations of muscle skeletal fibers and muscle injury with increased CK levels and can even cause rhabdomyolysis.^[8]^ Furthermore, other autoimmune disease including inflammatory myopathy and myasthenia gravis should be excluded when thyroid-related muscle damage is considered.

The speculation of relative hypothyroidism-induced myopathy was first described 20 years ago by Suzuki et al. Four adult patients with Grave diseases developed elevated CK levels with or without myalgia and muscle cramps, which occurred 2 to 8 weeks after the initiation of MMI despite no evidence of a hypothyroid state in blood and tissues during the treatment with antithyroid medications. The muscular symptoms were relieved within months regardless of whether there was a reduction of MMI dosage, as long as patients receive L-T4 therapy. In 1 particular case, the CK level was normalized after the administration of L-T4 and elevated again when the L-T4 dosage was halved without any changes of MMI dosage. Thus, rapid reduction of thyroid hormone or relative hypothyroidism was considered to be the cause for these myopathic changes, although the author could not exclude the possibility of muscle damage caused by side effects of the MMI.^[1]^ In recent years, several case reports supported the idea that myopathy can be induced by relative hypothyroid syndrome during the treatment of hyperthyroidism. As summarized in Table 1, all of the commonly used treatments for hyperthyroidism (including ATDs, thyroidectomy and 131-I treatment), can cause relative hypothyroidism syndrome. The muscular symptoms in most cases were developed within 4 to 8 weeks after the initiation the antithyroid treatment, and resolved by reduction/discontinuation of ATDs or administration of L-T4. The elevation of CK levels was always accompanied with a rapid reduction of thyroid hormone. Although in several cases thyroid hormone was mildly below the normal reference range, the TSH levels usually stayed suppressed, which was quite different to the typical hypothyroidism-induced myopathy. As summarized by Duyff et al, the mean TSH level was 53 mU/L in patients with hypothyroidism-induced myopathy.^[5]^ In particular, 1 patient who had received total thyroidectomy developed rhabdomyolysis only 2 weeks after the discontinuation of levo-triiodothyronine (L-T3) replacement in preparation for thyroid remnant ablation, suggesting that the rapid reduction of thyroid hormone cause more muscular damage than slowly progressed hypothyroidism.^[2]^

**Table 1:**
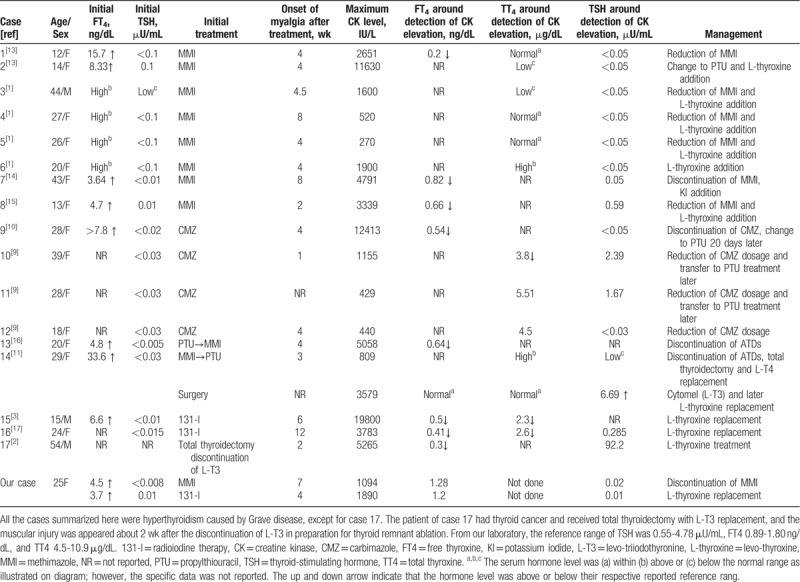
Summary of cases with CK elevation during the rapid decrease of serum thyroid hormone level.

However, in most early case reports, the muscle damages were tethered to their specific treatment such as MMI, carbimazole, propylthiouracil (PTU) or 131-I therapy. Such opinion is especially reasonable for patients treated with ATDs, because myopathy onset during treatment for hyperthyroidism with ATDs was once considered simply as a rare adverse effect of ATDs.^[9,10]^ And it was much difficult to prove whether the muscular damage was caused by particular treatment, or the relative hypothyroid syndrome, since most of patients only received 1 type of antithyroid treatment.

Our case report describes a patient respectively received ATDs and 131-I treatments, and developed muscular symptoms after each treatment accompanied by elevated muscle enzymes and normalization of thyroid hormone levels. Furthermore, the rapid relief of muscle symptoms was always associated to elevation of thyroid hormone. Because the patient has normal serum potassium and the concentrations of ANA, CRP, and ESR were within the normal range, and the muscular symptoms never relapsed during follow-up period, there was no evidence of diseases such as thyrotoxic periodic paralysis, auto-immune associated inflammatory myopathy, and myasthenia gravis. In addition, because the incidences of myopathy induced by both ATDs and 131-I treatment were extremely low, it was unlikely that this patient suffered 2 episodes of myopathy caused by these 2 treatments separately. The only case comparable to ours was reported by Shaheen et al. In that case, the patient with Graves’ disease developed myalgia 3 weeks after receiving MMI treatment and switching to PTU therapy did not resolve the muscular symptoms. The symptoms of muscle cramps and myalgia as well as CK level peaked several weeks after thyroidectomy when blood test showed normal thyroid hormone level with mild elevated TSH level (6.69 mIU/L) during L-T4 replacement therapy. The symptoms resolved and the CK level returned to normal range only 8 days after supplementation of L-T3.^[11]^ In summary, our particular case together with Shaheen's case can strongly support the idea of “relative hypothyroidism” induced myopathy during the treatment of hyperthyroidism.

The mechanisms underlying these myopathic changes remained unclear. As reviewed by Salvatore et al, thyroid hormone signaling regulates crucial biological functions in skeletal muscles, including energy expenditure, thermogenesis, development and growth and skeletal muscle repair. The intracellular T3 has 2 sources, which are T3 in serum that is secreted by thyroid gland, and T3 that is converted from T4 by type 2 intracellular iodothyronine deiodinase (DIO-2).^[12]^ Importantly, the expression and activity of DIO2 are tissue specific and could be modulated by different thyroid function. It has been reported that DIO2 mRNA levels or DIO2 activity in rodents’ skeletal muscle are increased in hypothyroidism state. Thus, we hypothesize that intracellular T3 equilibrium in skeletal muscles can exhibit adaptation by alteration the expression or activity of related receptors and enzymes in the state of hyperthyroidism. Rapid reduction of thyroid hormone could; therefore, result in muscle injury just like hypothyroidism. In addition, patient genetic backgrounds may also serve as risk factors because the majority of reported cases were Asians.

Patients complaints of onset of muscular weakness or myalgia after initiation antithyroid treatment should be treated as warning signals for relative hypothyroidism syndrome. In clinical practice, CK level and thyroid function should be closely monitored for such patients. When such syndrome is encounter, ATD dosage adjustment, or L-T4 replacement is recommended to ameliorate patient symptom. As suggested by Suzuki et al, hasty treatment of hyperthyroidism should be avoided unless the thyrotoxic conditions are critical or lethal, such as cardiac failure or thyrotoxic crisis.

## 4 Conclusion

In summary, we presented a patient with Graves’ disease who developed 2 episodes of myopathy induced by rapid correction of the hyperthyroid state or the so called “relative hypothyroidism” after 2 different treatments. Relative hypothyroidism might be overlooked as suggested in our review of literature. The mechanisms underlying these myopathic changes during antithyroid treatments should be further investigated.

## Author contributions

**Data curation:** Hongwei Gao.

**Funding acquisition:** Tianpei Hong.

**Resources:** Hongwei Gao.

**Supervision:** Hongwei Gao.

**Writing – original draft:** Ran Lu.

**Writing – review and editing:** Haining Wang, Tianpei Hong, Hongwei Gao.
